# MicroRNA‐210 Decreases heme Levels by Targeting Ferrochelatase in Cardiomyocytes

**DOI:** 10.1161/JAHA.113.000121

**Published:** 2013-04-24

**Authors:** Aijun Qiao, Arineh Khechaduri, R. Kannan Mutharasan, Rongxue Wu, Varun Nagpal, Hossein Ardehali

**Affiliations:** 1 Feinberg Cardiovascular Research Institute, Northwestern University, Chicago, IL (A.Q., A.K., K.M., R.W., V.N., H.A.)

**Keywords:** heme synthesis, hypoxia, miRNA, mitochondrion

## Abstract

**Background:**

MicroRNA‐210 (miR‐210) increases in hypoxia and regulates mitochondrial respiration through modulation of iron‐sulfur cluster assembly proteins (ISCU1/2), a protein that is involved in Fe/S cluster synthesis. However, it is not known how miR‐210 affects cellular iron levels or production of heme, another iron containing molecule that is also needed for cellular and mitochondrial function.

**Methods and Results:**

To screen for micro‐ribonucleic acids (miRNAs) regulated by iron, we performed a miRNA gene array in neonatal rat cardiomyocytes treated with iron chelators. Levels of miR‐210 are significantly increased with iron chelation, however, this response was mediated entirely through the hypoxia‐inducible factor (HIF) pathway. Furthermore, miR‐210 reduced cellular heme levels and the activity of mitochondrial and cytosolic heme‐containing proteins by modulating ferrochelatase (FECH), the last enzyme in heme biosynthesis. Mutation of the 2 miR‐210 binding sites in the 3′ untranslated region (UTR) of FECH reversed the miR‐210 response, while mutation of either binding site in isolation did not exert any effects. Changes mediated by miR‐210 in heme and FECH were independent of ISCU, as overexpression of an ISCU construct lacking the 3′ UTR does not alter miR‐210 regulation of heme and FECH. Finally, FECH levels increased in hypoxia, and this effect was not reversed by miR‐210 knockdown, suggesting that the effects of miR‐210 on heme are restricted to normoxic conditions, and that the pathway is overriden in hypoxia.

**Conclusions:**

Our results identify a role for miR‐210 in the regulation of heme production by targeting and inhibiting FECH under normoxic conditions.

## Introduction

Micro‐ribonucleic acids (miRNAs) are small 22 nucleotide RNA molecules that regulate gene expression by binding to the 3′ untranslated region (UTR) of messenger RNA (mRNA). It is predicted that miRNAs target and regulate about 30% of the mammalian genome.^1^ The role of miRNAs in several cardiac physiological and pathological processes has been characterized. These include cardiac development,^2^ cardiac hypertrophy,^3^ heart failure,^4^ and angiogenesis.^5^ miRNAs have both beneficial and detrimental effects in cardiac physiology.^6^

miRNA‐210 (miR‐210) levels have been shown to increase in response to hypoxia in various cell types, including cardiac cells. It is regulated by hypoxia‐inducible factor (HIF). However, recent evidence has demonstrated its regulation by p53‐ and AKT‐dependent pathways, as well. miR‐210 has been shown to possess diverse functions, including modulation of angiogenesis,^7^ stem cell survival,^8^ and hypoxia‐induced cell cycle arrest.^9^ In the heart, miR‐210 overexpression using minigenes in mice has been shown to improve cardiac function and reduce injury after myocardial infarction.^10^ Our group has also demonstrated that miR‐210 may have protective effects against oxidant‐induced cell death and reduce the production of reactive oxygen species in isolated cardiomyocytes. Furthermore, several reports have indicated that miR‐210 targets iron‐sulfur cluster scaffold proteins (ISCU 1/2),^11–13^ an important protein for the synthesis of iron‐sulfur clusters^14^ and consequently proper functioning of the electron transport chain.^15^ Through modulation of ISCU, miR‐210 has been shown to alter mitochondrial respiratory complex activity and mitochondrial function.^11–13^

Iron is an essential metal that is necessary for normal cellular processes, including metabolism, heme synthesis, cell proliferation, cytochrome p450 enzyme activity, and hypoxic response. Iron can quickly transform between a reduced ferrous (Fe^2+^) and oxidative ferric (Fe^3+^) state,^16^ a property which is important for many catalytic processes. However, this redox activity of iron can also lead to the production of hydroxyl radicals, the most toxic reactive oxygen species (ROS). Thus, the levels of iron are tightly regulated to provide sufficient amount of the molecule for important cellular processes while preventing iron‐mediated ROS production.

The 2 major iron containing molecules in the body are Fe/S clusters and heme. Both of these molecules are needed for mitochondrial respiration. The process of heme synthesis starts in the mitochondria, proceeds to synthesis of δ‐aminolevulinic acid, an intermediate molecule which is then transported to the cytoplasm for further enzymatic reactions and the synthesis of the porphyrin ring. The final and rate‐limiting step occurs in the mitochondria, where ferrochelatase (FECH) inserts an iron molecule into protoporphyrin IX to generate heme. FECH has been shown to be induced by hypoxia‐inducible factor (HIF),^17^ but its regulation by miRNAs is not known.

Here, we hypothesize that because of its role in mitochondrial respiration, miR‐210 might also regulate cellular iron levels and heme synthesis in rat cardiac cells. We show that miR‐210 levels are increased with iron chelation, but, this effect is entirely through a hypoxia‐inducible factor (HIF)‐dependent pathway. Modulation of miR‐210 alters cellular heme and the activity of both mitochondrial and cytosolic heme containing proteins. These effects occur through modulation of FECH by binding to the 3′ UTR of its respective mRNA molecule. Overexpression of FECH reverses the effects of miR‐210 on heme and FECH. miR‐210–mediated changes in heme and FECH are independent of ISCU, as overexpression of ISCU1/2 lacking the 3′ UTR does not alter miR‐210 response. These results provide a novel function for miR‐210 in altering cellular physiology in heart cells through modulation of cellular heme levels.

## Methods

### Cell Culture

H9c2 cardiac myoblasts were purchased from The American Type Culture Collection (ATCC) and kept in complete Dulbecco's modified Eagle medium (DMEM) (ATCC, Inc.) supplemented with 10% fetal bovine serum (FBS) (Invitrogen, Inc.) and 1% penicillin‐streptomycin (P/S). Human embryonic kidney cells (HEK293) cells were cultured in DMEM (CellGro, Mediatech, Inc.) supplemented with 10% FBS, penicillin‐streptomycin (P/S) and 5 mg/mL of sodium pyruvate. Wild‐type (WT) mouse embryonic fibroblasts (MEFs) as well as aryl hydrocarbon receptor nuclear translocator (ARNT) knockout (KO) MEFs were cultured in DMEM (CellGro) supplemented with 10% FBS, P/S, and 5 mg/mL of sodium pyruvate. Neonatal rat cardiomyocytes (NRCM) were prepared from 1‐ to 2‐day‐old Sprague‐Dawley rats as previously described.^18^

### Plasmids and Lentivirus Construction

The miR‐210 sensor construct used as a positive control was created as described previously.^19^ The WT 3′‐untranslated regions (UTRs) of FECH were cloned downstream of a firefly luciferase cassette in pMIR‐REPORT (Promega, Inc.). The mutant vectors of FECH 3′‐UTRs were constructed with a 4 nucleotide mismatch using the QuikChange Lightning Site‐Directed Mutagenesis Kit (Stratagene, Inc.). The open reading frame (ORF) of FECH with myc tag was amplified and cloned into the pCMV‐Script vector. An expression vector encoding the ORF of ISCU1 with a 3′ hemagglutinin (HA) tag (ISCU1‐HA) and an expression vector encoding the ORF of ISCU2 with a 3′ myc epitope (MYC) tag (ISCU2‐myc) were provided by Stephen Y. Chan (Brigham and Women's Hospital). All oligonucleotides were purchased from Integrated DNA Technologies. The primers used are shown in the Table 1. miR‐210 lentivector‐based anti‐miR‐210 (miRZip, a gift from Joseph C. Wu, Stanford University), as well as a nonsilencing control lentivector were transfected with the packaging plasmid psPAX2 and vesicular stomatitis virus glycoprotein G envelope plasmid pMD2.G into HEK293T cells by CaCl_2_ transfection for 3 days followed by collection of supernatant.

**Table 1.** Primers Used in Plasmid Construction and Real‐Time PCR

The primers for plasmid construction	
Rat FECH 3′UTR	F: 5′‐TTACTAGTCCCTGCCTGAGGACCCGTGG‐3′
	R: 5′‐TTAAGCTTTTCTGTTTGAAGAACAAAGTTTTAG‐3′
Rat FECH 3′UTR mut‐1	F: 5′‐GAGTTTCACATTTTTATCTTATGAGATACCGTGAGATTCTGCACTTATTGCCCAAAACAAC‐3′
	R: 5′‐GTTGTTTTGGGCAATAAGTGCAGAATCTCACGG TATCTCATAAGATAAAAATGTGAAACTC‐3′
Rat FECH 3′UTR mut‐2	F: 5′‐TTGAGCCCCAGCTGTGAACCGTGACATTATGTCACCGACTCG‐3′
	R: 5′‐CGAGTCGGTGACATAATGTCACGGTTCACAGCTGGGGCTCAA‐3′
Rat‐FECH‐overexpression plasmid‐myc tag	F: 5′‐GACCGAATTCACCATGCTTTCGGCCGGCGCCAA‐3′
	R: 5′‐TTACGTCGACTCACAGATCCTCTTCTGAGATGAGTTTTTGTTCCAGCTGTTGGCTGGTGAAGAAGG‐3′
The primers for real‐time PCR	
Rat‐ALAS1	F: 5′‐AACCGGAGAGCACAGATCTTCCC‐3′
	R: 5′‐ATAGTCGTTGCTGCACCAGACCG‐3′
Rat‐ALAS2	F: 5′‐ATAAGGGTGGGTAATGCGGCA‐3′
	R: 5′‐CTGTGCTTGGCGAGCAGAAGAT‐3′
Rat‐ALAD	F: 5′‐TGTTGTGGCACGGAGCCAAG‐3′
	R: 5′‐AATGTCAGCACCGGCTCTGC‐3′
Rat‐PGBD	F: 5′‐TGGCTGGCCTACAGCGCAT‐3′
	R: 5′‐TTCCTCTGGGTGCAAGATCTGG‐3′
Rat‐UROS	F: 5′‐TCGGTACTTAGCAGCCCCGGT‐3′
	R: 5′‐AAAGGTCCTCGCGGGCTAAGAG‐3′
Rat‐UROD	F: 5′‐ACAACAGCTGGCTGGACGTGTG‐3′
	R: 5′‐GTCATCAGGGTCCACGGAGCA‐3′
Rat‐FECH	F: 5′‐CCAATCGGGTCCAGCAGTGGT‐3′
	R: 5′‐CCCGTTTTGGGCTTCCTCCT‐3′
Rat‐CPO	F: 5′‐AGCTGAGGAGAGGGCGGTATGT‐3′
	R: 5′‐GCCAAACTTGGTGCCCCGAT‐3′
Rat‐PPO	F: 5′‐CTTGGACCTGAGGTGGCGTCT‐3′
	R: 5′‐CGGATGCTGAGCTCTTGGCT‐3′
Rat‐HOX1	F: 5′‐CAGAGGCCTGCTAGCCTGGTTC‐3′
	R: 5′‐GGATTTTCCTCGGGGCGTCT‐3′
Rat‐HOX2	F: 5′‐TGGCCCATGCTTACACTCGTTAC‐3′
	R: 5′‐CGCTGAGCCACCTTCTTCAGC‐3′
Rat‐ISCU	F: 5′‐TCCTTAGTGCAGTTGATGTCTTG‐3′
	R: 5′‐CCTGTCACTCTCCAGAGCTTAAG‐3′
Human‐ISCU1/2	F: 5′‐GCCAAGGAGCTCTGCCTTCCT‐3′
	R: 5′‐GCCAGCATGGAGCAGTGCAGT‐3′
Human‐FECH1	F: 5′‐CGTCACCACAGAAACAGCCCA‐3′
	R: 5′‐TTCCAGTTTTCGGCTTCCTGATG‐3′
Human‐FECH2	F: 5′‐CATGCCCAGGGTGCAAAACC‐3′
	R: 5′‐TTCGGCTTCCTCTTCTGCGG‐3′
18S	F: 5′‐AGTCCCTGCCCTTTGTACACA‐3′
	R: 5′‐CGATCCGAGGGCCTCACTA‐3′

PCR indicates polymerase chain reaction; FECH, ferrochelatase; UTR, untranslated region; ISCU, iron‐sulfur cluster assembly protein; ALAS, delta‐aminolevulinic acid synthase; ALAD, delta‐aminolevulinic acid dehydratase; PGBD, porphobilinogen deaminase; UROS, uroporphyrinogen III synthase; UROD, uroporphyrinogen III decarboxylase; CPO, coproporphyrinogen oxidase; PPO, protoporphyrinogen oxidase; HOX, heme oxygenase.

### Transfection

For miR‐210 overexpression experiments, control miRNA duplexes (Pre‐miR‐scr) and Pre‐miR‐210 miRNA precursors (Pre‐miR‐210) were purchased from Ambion, Inc. and transfected into H9c2 cells with Lipofectamine RNAiMAX Transfection Reagent (Invitrogen, Inc.). For miR‐210 rescue‐of‐function assays, H9c2 cells were transfected in 6 well plates with either ISCU1‐HA and ISCU2‐myc or enhanced green fluorescent protein (GFP) control plasmid (Stratagene, Inc.) (0.8 μg/well) along with either Pre‐miR‐210 or Pre‐miR‐scr (0.4 μg) according to the manufacturer's instructions of Transmessenger transfection reagent from Qiagen, Inc. All assays were conducted 48 hours later.

### Lentiviral Transduction of Cells

Virus was sterilely filtered (0.22 μm), and utilized for subsequent infection of H9c2 cells for gene transduction. The transduction of H9c2 cells was carried out in complete medium for 72 hours.

### MiRNA Array Profiling

The quality of the total RNA was verified by an Agilent 2100 Bioanalyzer profile. One thousand nanogram of total RNA from sample and reference was labeled with Hy3 and Hy5 fluorescent label using the miRCURY LNA array power labeling kit (Exiqon, Inc.) following the procedure described by the manufacturer. The Hy3‐labeled samples and a Hy5‐labeled reference RNA sample were mixed pair‐wise and hybridized to the miRCURY LNA Array version 5th Generation (Exiqon, Inc.), which contains capture probes targeting all miRNAs for human, mouse, or rat registered in the miRBASE version 16.0 at the Sanger Institute. The hybridization was performed according to the miRCURY LNA array manual using a Tecan HS4800 hybridization station (Tecan Group Ltd.). After hybridization the microarray slides were scanned and stored in an ozone free environment (ozone level below 2.0 ppb) in order to prevent potential bleaching of the fluorescent dyes. The miRCURY LNA array microarray slides were scanned using the Agilent G2565BA Microarray Scanner System (Agilent Technologies, Inc.) and the image analysis was carried out using the ImaGene 9.0 software (BioDiscovery, Inc.). The quantified signals were background corrected (Normexp with offset value 10) and normalized using the global LOWESS (LOcally WEighted Scatterplot Smoothing) regression algorithm.^20^

### Quantitative Real‐Time Polymerase Chain Reaction

Total cellular RNA was isolated using RNA‐STAT‐60 reagent (TEL‐TEST, Inc.) according to the manufacturer's protocol. For mRNA expression analysis, total RNA was reverse transcribed into cDNA using M‐MLV Reverse Transcriptase (Applied Biosystems, Inc.) and analyzed using the SYBR Green Polymerase Chain Reaction (PCR) MasterMix (Applied Biosystems, Inc.) on a 7500 Real‐Time PCR System (Applied Biosystems, Inc.) using standard conditions. The fold changes of mRNA expression were quantified with the 2^−ΔΔCt^ relative quantification method using 18S as the normalization control. The primers used are shown in Table 1. Taqman miRNA assays (Applied Biosystems, Inc.) were performed to quantify miR‐210 levels. RNA species‐specific cDNA templates were generated according to manufacturer protocols for both miR‐210 and U6, a small RNA which served as a normalization control. miR‐210 expression relative to U6 control was calculated using the 2^−ΔΔCt^ method. The reagents for the assay, including primers for miR‐210 and U6 control were purchased from Applied Biosystems, Inc.

### Luciferase Assays

Luciferase assays were performed in H9c2 cells after cotransfection of RNA duplexes and plasmids in the following combinations along with the pRL‐TK Renilla plasmid for normalization: (1) pre‐miR‐210/FECH 3′‐UTR plasmid, (2) pre‐miR‐scr/FECH 3′‐UTR plasmid, (3) positive control plasmid, (4) negative control plasmid (empty pMIR‐REPORT vector). Luminescence was measured using a Berthold Lumat LB 9507 luminometer with the Dual‐Luciferase Assay Kit (Promega, Inc.). For miR‐210 knockdown experiments, control miRNA duplexes (anti‐miR‐scr) and Anti‐miR–210 miRNA inhibitors (anti‐miR–210) were purchased from Ambion, Inc.

### Western Blot

Cells were lysed using RIPA buffer (Thermo Fisher Scientific, Inc.) with added protease inhibitor cocktail (G‐Biosciences, Inc.). Thirty μg total protein were loaded on a 4% to 12% SDS‐PAGE gradient gel (Invitrogen, Inc.), transferred to a nitrocellulose membrane, incubated with rabbit FECH or ISCU antibody (Proteintech Group, Inc.) at 1000‐fold dilution, then incubated with horseradish peroxidase‐conjugated goat anti‐rabbit secondary antibody (Santa Cruz Biotechnology, Inc.). Enhanced chemiluminescence (ECL) was then detected (Thermo Fisher Scientific, Inc.). The membrane was then stripped and anti‐GAPDH antibody (Santa Cruz Biotechnology, Inc.) was used as a loading control. The rabbit anti‐c–Myc antibody was purchased from Abcam, Inc.

### Heme Content Determination

For determination of cellular heme levels, cells were lysed in 1% Triton‐X100 in phosphate buffered saline (PBS), followed by centrifugation at 5000*g* for 10 minutes to remove debris. Protein concentration was quantified by bicinchoninic acid (BCA) assay (Thermo Fisher Scientific, Inc.) and heme was quantified as described.^21^ Briefly, equal amounts of protein were mixed with 2 mol/L oxalic acid, heated to 95°C for 30 minutes to release iron from heme and generate protoporphyrin IX. Samples were then centrifuged for 10 minutes at 1000*g* at 4°C to remove debris. The fluorescence of the supernatant was assessed at 405/600 nm on Spectra Max Gemini fluorescence microplate reader and normalized to protein concentration of each sample.

### Iron Content Determination

Cellular iron levels were measured with iron assay kit (Biovision, Inc.) according to the manufacturer's instructions. Briefly, the cells from 6 well plates were lysed in 65 μL iron assay buffer, centrifuge at 16 000*g* for 10 minutes to remove insoluble materials. Fifty microliters of the supernatant was used to measure absorbance at 560 nm, and the results were normalized to protein concentration of each sample.

### Enzyme Activities

Complex IV activity was measured using the Sandwich ELISA Kits–Microplate assay (MitoSciences, Inc.) according to the manufacturer's protocol. Peroxidase activity was assessed with the Amplex Red Hydrogen Peroxide/Peroxidase Assay Kit (Invitrogen, Inc.) as absorbance at 560 nm and normalized to protein concentration of each sample.

### Hypoxia

All hypoxia experiments were conducted in a hypoxia glove box (Coy Laboratory Products, Inc.).

### Statistical Methods

Data are reported as mean±standard error (SE). Significance threshold was set at *P*=0.05, and, because the data evaluated may reasonably be assumed to be normally distributed, the Student *t*‐test was used to assess statistical significance for all comparisons, except for Figures 9C, 11A, and 11B, where 2‐way analysis of variance (ANOVA) with Tukey post hoc analysis was used.

## Results

### miR‐210 Levels Are Increased in Response to Iron Chelation

**Figure 1.**
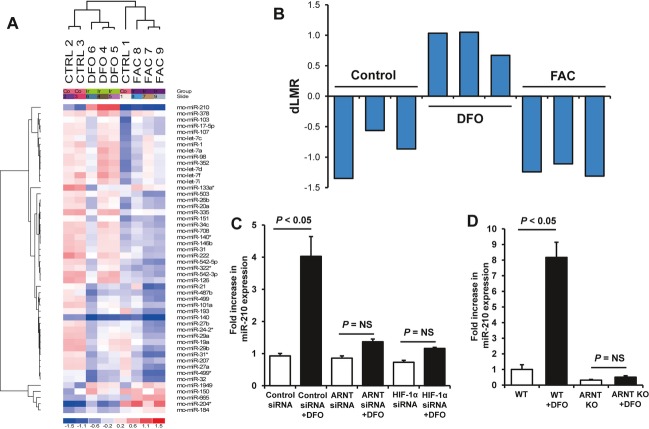
DFO increases miR‐210 levels through a HIF‐dependent pathway. A, Heatmap plot of microRNA expression in response to FAC and DFO in neonatal rat cardiomyocytes. B, Difference in log median ratios (DLMR) for miR‐210 in response to DFO and FAC; n=3 in each group. C, Changes in miR‐210 levels in NRCM treated with DFO in the presence and absence of ARNT or HIF‐1α siRNA. D, Changes in miR‐210 in wild type and ARNT knockout MEFs in response to DFO. There is no increase in the levels of miR‐210 after DFO treatment in ARNT knockout MEFs. Data are presented as mean±standard error of the mean (SEM). *P<0.05, n=6 in each group for (C) and (D). DFO indicates desferoxamine; HIF, hypoxia‐inducible factor; FAC, ferric ammonium citrate; NS, nonsignificant; siRNA, small interfering RNA; NRCM, neonatal rat cardiomyocytes; ARNT, aryl hydrocarbon receptor nuclear translocator; MEF, mouse embryonic fibroblast; KO, knockout.

miR‐122 has been shown to be regulated by systemic iron levels^22^. However, it is not known how cellular iron alters miRNA levels. In order to identify miRNAs that are altered in response to cellular iron overload or chelation, we treated NRCM with 0.25 mmol/L of desferoxamine (DFO, an iron chelator) or 50 μg/mL of ferric ammonium citrate (FAC) for 24 hours. miR‐210 levels were significantly altered in response to DFO, while the addition of FAC only resulted in a modest change in some miRNAs (Figure 1A and 1B). Other miRNAs did not show any significant or only a modest change. In order to better characterize the role of miR‐210 in iron chelation, we then performed quantitative real‐time PCR, which demonstrated that miR‐210 levels were increased by ≈4‐ and 8‐fold with DFO treatment in NRCM and MEFs, respectively (Figure 1B and 1C). Since DFO is known to also stimulate HIF, we assessed whether deletion of the HIF pathway would have an effect on the increase in miR‐210 in response to DFO. HIF‐1α and ‐2α dimerize with ARNT to bind DNA and deletion of ARNT leads to complete inactivation of the HIF pathway. Knockdown of HIF‐1α or ARNT eliminated the response to DFO in NRCM (Figure 1B). Furthermore, MEF with deletion of ARNT displayed almost complete attenuation of the response to DFO (Figure 1C). These data suggest that the effects of DFO are almost exclusively caused by the activation of HIF, and not because of direct effects of iron. Since miR‐210 has been shown to be activated by hypoxia and HIF, these results indicate that changes in cellular iron likely result in no major effect on miRNA profile. This is in contrast to the systemic iron regulation, which has been shown to be regulated by miR‐122.^22^

### miR‐210 Reduces Cellular Heme and Heme‐Containing Proteins

**Figure 2.**
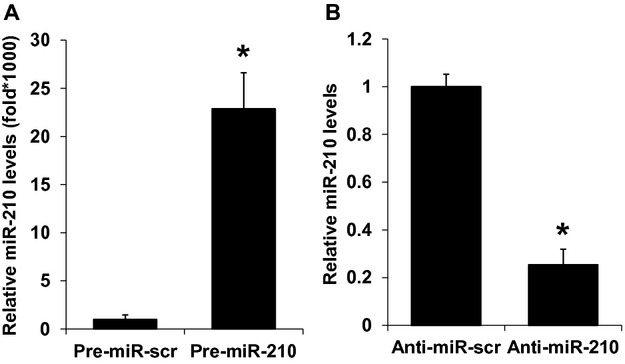
Modulation of miR‐210 in H9c2 cells. A, miR‐210 levels after transfection of H9c2 cells with pre‐miR‐210. B, miR‐210 levels after transfection with anti‐miR‐210. Data are presented as mean±standard error of the mean (SEM). *P<0.05, n=6 in each group.

**Figure 3.**
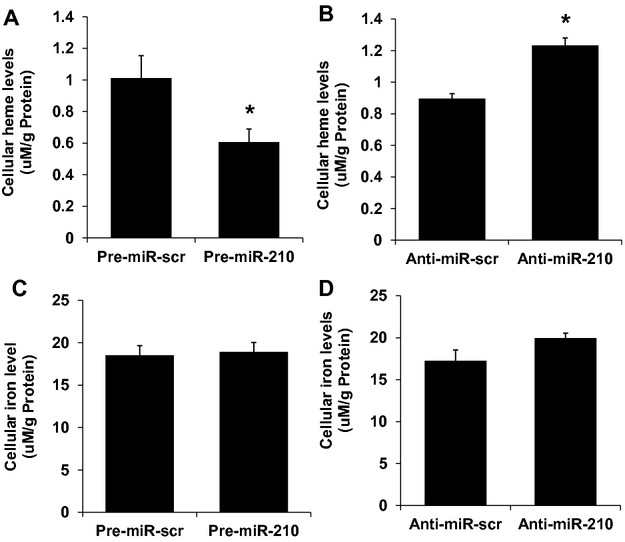
miR‐210 influences the levels of heme in H9c2 cells. Heme levels are decreased with miR‐210 overexpression (A), while its levels are increased in response to miR‐210 downregulation (B). Cellular levels of iron are not altered in response to miR‐210 overexpression (C) or downregulation (D). Data are presented as mean±standard error of the mean (SEM). *P<0.05, n=5 in each group.

miR‐210 has been shown to target ISCU and decrease Fe/S cluster levels, but its role in the regulation of another iron‐containing molecule, heme, is not known. We next hypothesized that miR‐210 regulates heme levels in cardiomyocytes. To address this hypothesis, we modulated the levels of miR‐210 in H9c2 cardiomyoblasts (Figure 2A and 2B), and then measured the cellular heme levels. It should be noted that miR‐210 is expressed at basal levels in H9c2 cells (Figure 2B), allowing us to downregulate its levels with an RNA‐interference approach. Overexpression and downregulation of miR‐210 in H9c2 cells resulted in a decrease and increase in heme levels, respectively (Figure 3A and 3B). The changes in heme levels are not due to the alteration of cellular iron, as overexpression of miR‐210 had no effect on iron levels (Figure 3C and 3D).

**Figure 4.**
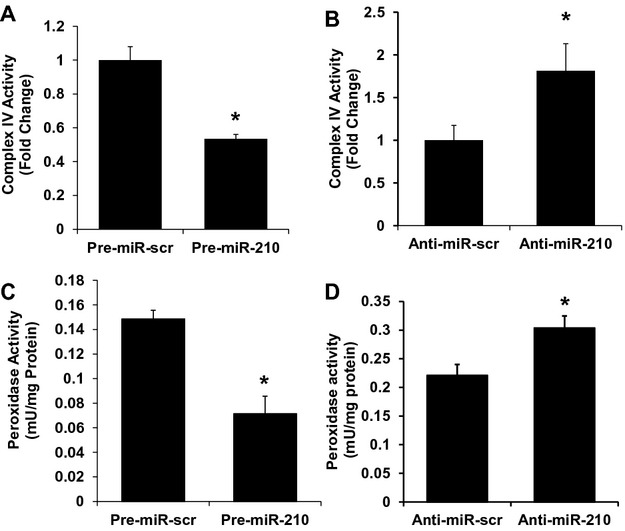
miR‐210 alters the activity of heme‐containing proteins. Mitochondrial complex IV activity is decreased or increased in response to miR‐210 overexpression (A) or downregulation (B). The activity of peroxidase is decreased with miR‐210 overexpression (C), while its levels are increased in response to miR‐210 downregulation (D). Data are presented as mean±standard error of the mean (SEM). *P<0.05, n=6 in each group.

We then assessed whether the regulation of heme levels by miR‐210 leads to changes in the activity of heme‐containing proteins. Overexpression of miR‐210 in H9c2 cardiomyoblasts resulted in a significant decrease in the activity of a mitochondrial (complex IV) and a cytoplasmic (peroxidase) heme‐containing protein, while its downregulation had the opposite effect (Figure 4A through 4D). Thus, miR‐210 decreases heme levels, leading to a decrease in the activity of heme containing proteins.

### miR‐210 Decreases the Levels of FECH

**Figure 5.**
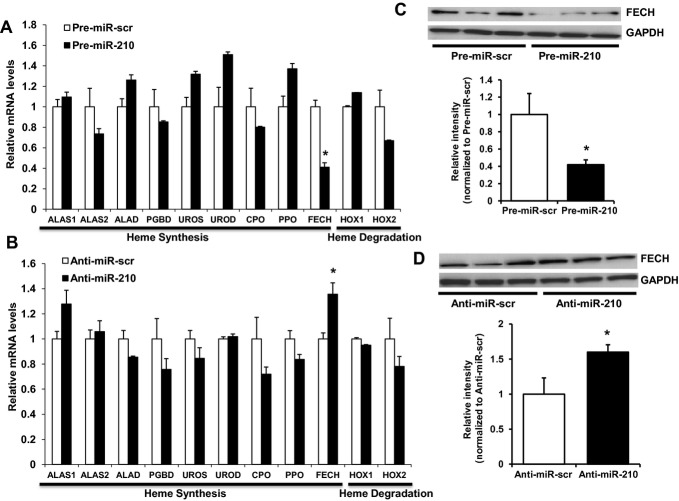
Among genes involved in heme synthesis or degradation, only FECH mRNA and protein levels are altered in response to miR‐210 modulation. mRNA levels of genes involved in heme synthesis or degradation, as assessed by RT‐PCR, in response to miR‐210 overexpression (A) or downregulation (B). miR‐210 inhibits FECH protein by targeting the 3′ UTR of its respective mRNA. FECH protein levels are reduced in response to miR‐210 overexpression (C), while they are increased with miR‐210 downregulation (D). Summary bar graphs are shown below the Western blots. Data are presented as mean±standard error of the mean (SEM). *P<0.05, n=3 in each group. FECH indicates ferrochelatase; RT‐PCR indicates reverse transcription polymerase chain reaction; UTR, untranslated region; ISCU, iron‐sulfur cluster assembly protein.

We then assessed the mechanism for the regulation of heme in response to miR‐210 modulation by measuring the mRNA levels of genes involved in heme synthesis and degradation. FECH, 1 of the 2 rate limiting enzymes in heme synthesis, carries out the last enzymatic reaction by inserting iron into protophorphyrin‐IX to make heme.^23^ Among all proteins involved in heme synthesis and degradation, FECH was the only protein whose mRNA was decreased in response to miR‐210 overexpression, while miR‐210 downregulation had the opposite effect (Figure 5A and 5B). To confirm these results, we assessed the levels of FECH protein with modulation of miR‐210. miR‐210 overexpression and downregulation resulted in a decrease and an increase in FECH protein levels, respectively (Figure 5C and 5D). Thus, miR‐210 inhibits cellular heme by reducing the levels of FECH.

**Figure 6.**
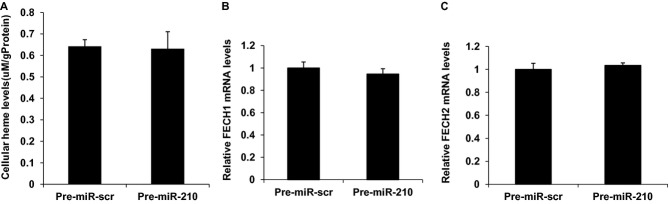
miR‐210 does not alter the levels of heme or FECH in HEK293 cells. A, Cellular heme levels after transfection of HEK293 cells with pre‐miR‐210. B and C, FECH levels after transfection of HEK293 cells with pre‐miR‐210; n=6 in each group. FECH indicates ferrochelatase; HEK, human embryonic kidney cells.

To determine whether the effects of miR‐210 on heme and FECH are also present in cells from other species, we conducted similar studies in HEK293 cells. Unlike rodent cells, manipulation of miR‐210 in HEK293 cells did not alter either the heme or FECH mRNA levels (Figure 6A through 6C), suggesting that the effects of miR‐210 on heme levels are unique to rodent cells and are not evolutionarily conserved in humans.

### miR‐210 Targets the 3′ UTR of FECH

**Figure 7.**
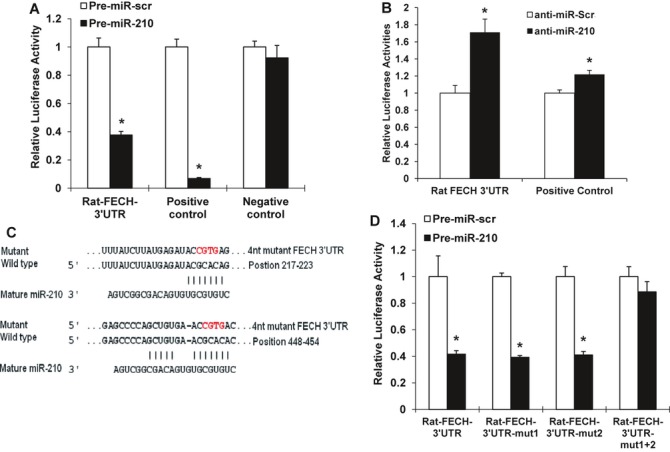
FECH mRNA is a direct target of miR‐210. A, miR‐210 overexpression in H9c2 cells inhibits luciferase activity of a construct containing the 3′ UTR of FECH. Positive control include 2 perfect matches to miR‐210 and negative control use pMIR‐report empty plasmid. B, miR‐210 downregulation results in an increase in luciferase activity. C, Schematic representation of the 2 putative miR‐210 binding sites in the FECH mRNA 3′ UTR. The site that was mutated is “GCAC.” D, While mutation of each site individually did not later the FECH response to miR‐210, mutation of both sites completely ameliorated the effect. Data are presented as mean±standard error of the mean (SEM). *P<0.05, n=6 in each group. FECH indicates ferrochelatase; UTR, untranslated region.

To determine whether FECH mRNA is a direct target of miR‐210, we engineered the 3′ UTR of the rat FECH into a luciferase construct, and then measured luciferase activity with miR‐210 modulation. As shown in Figure 7A and 7B, miR‐210 overexpression resulted in a significant decrease in luciferase activity, while its downregulation had the opposite effect. Upon further examination of the rat FECH 3′ UTR, we identified 2 putative miR‐210 binding sites (Figure 7C). We then assessed the relative role of these 2 binding sites on miR‐210 targeting. Mutation of each of the sites did not alter miR‐210‐mediated reduction in luciferase activity. However, mutation of both sites completely abolished the miR‐210 effects (Figure 7D).

### FECH Mediates the Effects of miR‐210 on Cellular Heme

**Figure 8.**
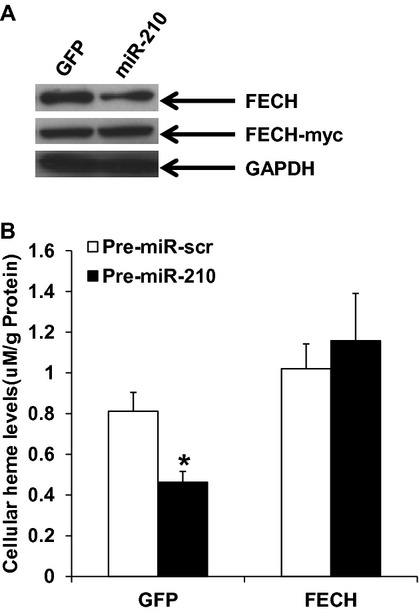
miR‐210 targeting of FECH is responsible for the changes in cellular heme levels. A, While the endogenous FECH protein levels are reduced with miR‐210 overexpression, the myc‐tagged protein lacking the 3′ UTR is unresponsive. B, Overexpression of FECH (lacking the 3′ UTR) does not lead to a reduction in cellular heme levels. Data are presented as mean±standard error of the mean (SEM). *P<0.05, n=4 to 6 in each group. FECH indicates ferrochelatase; UTR, untranslated region; GFP, green fluorescent protein.

We next assessed whether miR‐210 targeting of FECH is responsible for the changes in cellular heme levels. A construct containing a myc‐tagged FECH without the 3′ UTR was transfected into H9c2 cells. While miR‐210 overexpression decreased the levels of the endogenous FECH, it had no effect on the myc‐tagged protein (Figure 8A), confirming that the removal of the 3′ UTR of FECH results in insensitivity to miR‐210 modulation. We then assessed the effects of overexpression of truncated FECH on cellular heme levels. While overexpression of miR‐210 reduced heme levels in GFP‐tranfected control cells, it did not have any effect on cells with overexpression of the FECH construct lacking the 3′ UTR (Figure 8B). These results demonstrate that FECH mediates the effects of miR‐210 on cellular heme levels.

### The Effect of miR‐210 on FECH is Not Through ISCU

**Figure 9.**
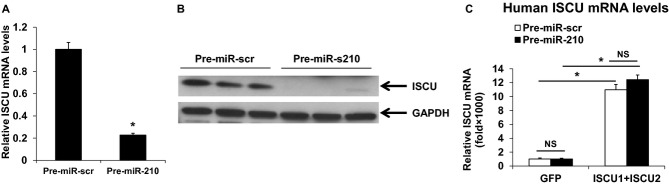
Overexpression of ISCU lacking the 3′ UTR does not alter the effects of miR‐210 on FECH or heme. ISCU mRNA (A) and protein (B) levels in response to miR‐210 overexpression in H9c2 cells. Human ISCU (C) levels are significantly increased after cotransfection of miR‐210 and human ISCU. Data are presented as mean±standard error of the mean (SEM). *P<0.05, n=3 to 6 in each group. Two‐way ANOVA with Tukey post hoc analysis was used for (C). ISCU indicates iron‐sulfur cluster assembly protein; FECH, ferrochelatase; UTR, untranslated region; ANOVA, analysis of variance; GFP, green fluorescent protein.

Since FECH contains an Fe/S cluster, it may be argued that the effects of miR‐210 on FECH may be due to a reduction in Fe/S cluster availability because of ISCU inhibition. We next assessed the role of ISCU in miR‐210–mediated changes in the levels of FECH. Consistent with previous findings,^11–13^ overexpression of miR‐210 resulted in a decrease in the levels of ISCU mRNA and protein (Figure 9A and 9B). We then transfected H9c2 cells with an ISCU construct lacking the 3′ UTR, rendering it unresponsive to miR‐210 (Figure 9C).^11^ Treatment of cells with pre‐miR–210 increased the levels of miR‐210 irrespective of ISCU overexpression (202±42 for GFP transfected cells and 827±129 for ISCU1+ISCU2 transfected cells).

**Figure 10.**
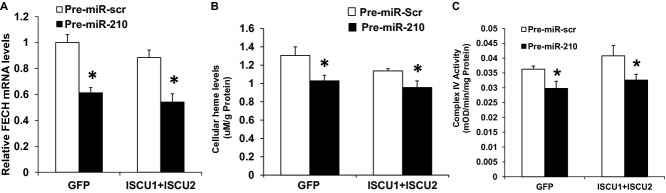
Effects of ISCU overexpression on FECH and heme levels in response to miR‐210. A, FECH mRNA levels are reduced with miR‐210 in the presence and absence of overexpression of ISCU construct lacking the 3′ UTR. B, The response of cellular heme levels to miR‐210 is not altered with overexpression of ISCU construct lacking the 3′ UTR. C, The reduction in complex IV activity with miR‐210 is not reversed by overexpression of the truncated ISCU1/2. Data are presented as mean±standard error of the mean (SEM). *P<0.05, n=3 to 5 in each group. ISCU indicates iron‐sulfur cluster assembly protein; FECH, ferrochelatase; UTR, untranslated region; GFP, green fluorescent protein.

We then assessed the effects of ISCU overexpression on FECH and heme levels in response to miR‐210. If miR‐210‐mediated effects on FECH are independent of ISCU, it would be expected that overexpression of the truncated ISCU1/2 constructs does not reverse changes in FECH and heme levels in response to miR‐210. As shown in Figure 10A and 10B, miR‐210 led to a decrease in FECH and heme levels were maintained even with ISCU1/2 overexpression. Furthermore, the effects of miR‐210 on complex IV activity were not reversed with overexpression of the truncated ISCU1/2 (Figure 10C). These results indicate that the effects of miR‐210 on FECH and heme are independent of ISCU and through direct targeting of FECH mRNA by miR‐210.

### The Increase in FECH Levels in Response to Hypoxia is Not Reversed by miR‐210

**Figure 11.**
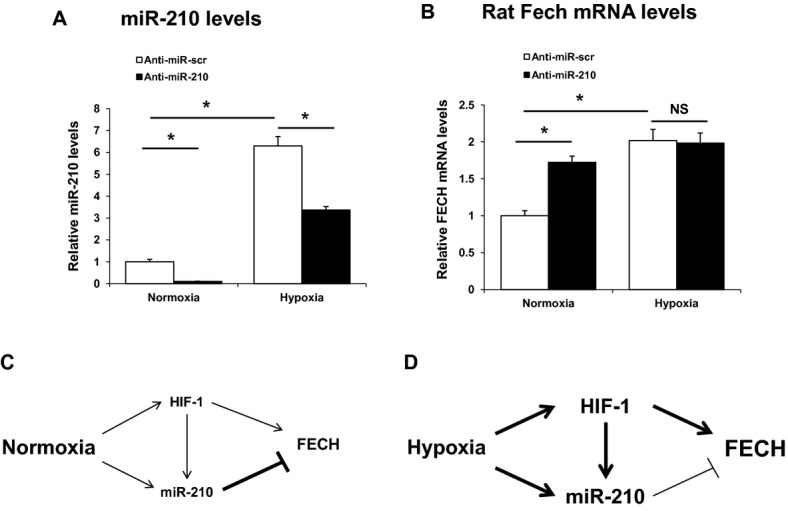
The increase in FECH levels in response to hypoxia is not reversed by miR‐210. A, miR‐210 levels are increased in response to 1.0% oxygen for 12 hours in H9c2 cells and the effect is reversed with miR‐210 downregulation. B, FECH levels are increased in response to hypoxia, however, the increase is not reversed with miR‐210 knockdown. C and D, Proposed model for the effects of HIF and miR‐210 on FECH levels in response to hypoxia. In normoxia the miR‐210 regulation of FECH predominates (C) whereas in hypoxia the direct effects of HIF‐1 on FECH induction predominate (D). Data are presented as mean±standard error of the mean (SEM). *P<0.05, n=5 to 6 in each group. Two‐way ANOVA with Tukey post hoc analysis was used for (A) and (B). FECH indicates ferrochelatase; HIF, hypoxia‐inducible factor; ANOVA, analysis of variance.

Previous studies have shown that HIF pathway induces the expression of FECH.^17^ Thus, there appear to be 2 pathways that modulate the levels of FECH in hypoxia: its induction by the HIF pathway and its inhibition through miR‐210. We next studied the overall effects of hypoxia on FECH levels in H9c2 cells. Hypoxia significantly increased the levels of miR‐210, and this effect was reversed by downregulation of miR‐210 (Figure 11A), consistent with previous reports.^7,11–13,19^ FECH levels also increased in response to hypoxia, however, this effect was not reversed by miR‐210 knockdown (Figure 11B). These results suggest that while in normoxia the predominant mode of FECH regulation is by miR‐210, in hypoxia the direct effects of HIF on FECH levels predominate, and miR‐210 regulation of FECH is overridden (Figure 11C and 11D).

## Discussion

In this article, we assessed changes in cellular miRNAs in response to iron overload and chelation. We also hypothesized that, in addition to the regulation of ISCU, miR‐210 modulates the expression of heme, and these 2 regulatory pathways together modulate mitochondrial function. We demonstrate that treatment with DFO in H9c2 cells results in an increase in miR‐210 levels, however, this effect is entirely mediated through the HIF pathway. miR‐210 regulates heme levels and the activity of heme‐containing proteins by modulating FECH through binding to its consensus sequences in the 3′ UTR of FECH. These effects are independent of ISCU, as overexpression of ISCU does not alter the miR‐210–mediated changes in FECH and heme. Thus, we propose that in cardiac cells, miR‐210 alters mitochondrial function through 2 pathways: (1) modulation of ISCU and production of Fe/S clusters, which are needed for several mitochondrial respiratory complexes, and (2) inhibition of FECH and reducing heme production, which is also needed for the activity of mitochondrial respiratory proteins.

Although the 3′ UTR of rat FECH mRNA has the consensus sequence for 2 miR‐210 binding sites, the human 3′ UTR is significantly longer and lacks any putative sites for miR‐210 binding. Furthermore, miR‐210 does not alter heme and FECH levels in human cells. This suggests that there has been divergence between rat and human in that humans lost the ability to modulate cellular respiration through changes in heme levels by miR‐210. This could be due to a redundancy in the miR‐210 effects through ISCU‐ and heme‐mediated pathways, or that in more advanced organisms, the regulation of heme became extinct and the ISCU‐mediated mechanism became the sole pathway in the regulation of mitochondrial function by miR‐210. Heme is a major component of hemoglobin, which plays a major role in the circulatory system and the delivery of blood to the peripheral tissue. Thus, it is possible that in humans, the regulatory effects of miR‐210 on heme were lost in order to ensure that heme is available and does not become a limiting factor for hemoglobin production under hypoxic conditions.

It has previously been shown that miR‐210 regulates mitochondrial function through modulation of ISCU and the resultant Fe/S cluster production. Here, we demonstrate another pathway by which miR‐210 regulates mitochondrial function. This pathway targets the production of another cofactor that is required for the activity of mitochondrial complexes, heme. Although both of these molecules contain iron, the levels of iron did not change in response to miR‐210 in our studies. Iron is an essential molecule and its cellular levels are regulated by iron regulatory proteins (IRP1/2). At the systemic level, iron is regulated by the protein hepcidin, although recent studies have demonstrated a role for miR‐122 in systemic iron regulation, as well. So far, no miRNAs have been identified to regulate cellular iron levels and our results demonstrate that changes in iron do not affect the miRNA profile. Since iron is an evolutionarily ancient molecule, and miRNA regulation appears to be absent in prokaryotic cells, it is possible that cellular iron levels were spared from miRNA regulation in eukaryotic cells. Instead, miRNA regulation of iron‐requiring processes appears to be at the level of individual pathways (ie, heme and Fe/S cluster synthesis).

In addition to miR‐210, the HIF pathway is also activated in response to hypoxia. These 2 pathways play a major role in the regulation of gene expression under hypoxic conditions. Our results demonstrated that the regulation of heme production by miR‐210 is no longer active under hypoxic conditions, suggesting that this phenomenon is only present when ample oxygen is available. Previous reports on miR‐210 have mostly indicated that the effects of this miRNA are under hypoxic conditions. Our results provide an alternative system in which the majority of miR‐210 effects are under normoxic conditions, and the system is overridden when oxygen becomes scarce. Recent studies have shown that HIF pathway plays a major role in the heart under normoxic conditions,^24–25^ in addition to its primary effects in hypoxia. A similar system may also exist with miR‐210, as shown in our paper and previous reports on this miRNA.

In conclusion, we demonstrate that miR‐210 regulates heme levels and the activity of heme‐containing proteins in rat cardiac cells through modulation of FECH and independent of ISCU.
